# Correction to: Laser-assisted surgery of the upper aero-digestive tract: a clarification of nomenclature. A consensus statement of the European Laryngological Society

**DOI:** 10.1007/s00405-017-4851-x

**Published:** 2018-01-06

**Authors:** Marc Remacle, Christoph Arens, Mostafa Badr Eldin, Guillermo Campos, Carlos Chiesa Estomba, Pavel Dulguerov, Ivana Fiz, Anastasios Hantzakos, Jerôme Keghian, Francesco Mora, Nayla Matar, Giorgio Peretti, Cesare Piazza, Gregory N. Postma, Vyas Prasad, Elisabeth Sjogren, Frederik G. Dikkers

**Affiliations:** 1 Department of Otorhinolaryngology, University Hospitals Magdeburg, Otto-von-Guericke University, Magdeburg, Germany; 2 Department ENT-HNS, Ain-Shams University, Cairo, Egypt; 3 Department of Surgery, Fundación Santa Fe Hospital Universitario, Instituto de Laringología, Universidad de la Sabana School of Medicine, Bogotá, DC Colombia; 4 ENT-Head and Neck Department, Hospital Universitario Donostia, San Sebastián, Spain; 5 Department of Otorhinolaryngology, Head and Neck Surgery, Academic Medical Center, University of Amsterdam, Amsterdam, The Netherlands; 6 Department of Otorhinolaryngology, Head and Neck Surgery, Geneva University Hospital, Geneva, Switzerland; 7 Department of Otorhinolaryngology, Head and Neck Surgery, Katharinenhospital, Stuttgart, Germany; 8 Department of Otolaryngology, Head and Neck Surgery, Surgical Subspecialties Institute, Cleveland Clinic Abu Dhabi, Abu Dhabi, United Arab Emirates; 9 Department of Otorhinolaryngology Head and Neck Surgery, CHL-Eich, Rue d’Eich 78, Luxembourg, Luxembourg; 10 Faculty of Medicine, Hotel Dieu de France Hospital, Saint Joseph University, Beirut, Lebanon; 11 Department of Otorhinolaryngology Head and Neck Surgery, University of Genoa, Genoa, Italy; 12 Department of Otorhinolaryngology, Head and Neck Surgery, University of Brescia, Brescia, Italy; 13 Department of Otolaryngology, Center for Voice, Airway and Swallowing Disorders, Medical College of Georgia at Augusta University, Augusta, GA USA; 14 Department of Otolaryngology Head and Neck Surgery, National University Health System, Ng Teng Fong General Hospital, Singapore, Singapore; 15 Department of ENT, Head and Neck Surgery, Leiden University Medical Center, Leiden, The Netherlands

## Correction to: Eur Arch Otorhinolaryngol (2017) 274:3723–3727 10.1007/s00405-017-4708-3

The article ‘Laser-assisted surgery of the upper aero-digestive tract: a clarification of nomenclature. A consensus statement of the European Laryngological Society,’ written by Marc Remacle, Christoph Arens, Mostafa Badr Eldin, Guillermo Campos, Carlos Chiesa Estomba, Pavel Dulguerov, Ivana Fiz, Anastasios Hantzakos, Jerôme Keghian, Francesco Mora, Nayla Matar, Giorgio Peretti, Cesare Piazza, Gregory N. Postma, Vyas Prasad, Elisabeth Sjogren, Frederik G. Dikkers, was originally published Online First without open access. After publication in volume 274 issue 10, page 3723–3727 the authors decided to opt for Open Choice and to make the article an open access publication. Therefore, the copyright of the article has been changed to © The Author(s) 2018 and the article is forthwith distributed under the terms of the Creative Commons Attribution 4.0 International License (http://creativecommons.org/licenses/by/4.0/), which permits use, duplication, adaptation, distribution and reproduction in any medium or format, as long as you give appropriate credit to the original author(s) and the source, provide a link to the Creative Commons license, and indicate if changes were made.

